# A Year of Critical Care: The Changing Face of the ICU During COVID-19

**DOI:** 10.14797/mdcvj.1041

**Published:** 2021-12-15

**Authors:** Atiya Dhala, Deepa Gotur, Steven Huan-Ling Hsu, Aditya Uppalapati, Marco Hernandez, Jefferson Alegria, Faisal Masud

**Affiliations:** 1 Houston Methodist Hospital, Houston, Texas, US; 2 Houston Methodist DeBakey Heart & Vascular Center, Houston Methodist Hospital, Houston, Texas, US; 3 J C Walter Jr Transplant Center, Houston, Texas States

**Keywords:** SARS-CoV-2, COVID-19, ICU, pandemic response, critical care, tele-critical care, bed capacity, staffing, PPE, burnout

## Abstract

During the SARS-CoV-2 pandemic, admissions to hospital intensive care units (ICUs) surged, exerting unprecedented stress on ICU resources and operations. The novelty of the highly infectious coronavirus disease 2019 (COVID-19) required significant changes to the way critically ill patients were managed. Houston Methodist’s incident command center team navigated this health crisis by ramping up its bed capacity, streamlining treatment algorithms, and optimizing ICU staffing while ensuring adequate supplies of personal protective equipment (PPE), ventilators, and other ICU essentials. A tele–critical-care program and its infrastructure were deployed to meet the demands of the pandemic. Community hospitals played a vital role in creating a collaborative ecosystem for the treatment and referral of critically ill patients. Overall, the healthcare industry’s response to COVID-19 forced ICUs to become more efficient and dynamic, with improved patient safety and better resource utilization. This article provides an experiential account of Houston Methodist’s response to the pandemic and discusses the resulting impact on the function of ICUs.

## Introduction

The SARS-CoV-2 pandemic has brought seismic changes to the healthcare industry, forcing it to adapt and innovate with every new coronavirus disease 2019 (COVID-19) surge. Specifically, challenges posed by the pandemic have altered the conventional approach to managing both intensive care units (ICUs) and ICU patients. This article offers an experiential account of critical care services at Houston Methodist during the COVID-19 pandemic and discusses the paradigm shifts in ICU operations and the delivery of critical care.

The initial outbreak of the virus in China and its early spread to Italy and Iran found both local and global healthcare organizations ill equipped to address the disease’s scale and scope.^1^ Treatment for COVID-19 patients was largely limited to supportive care since there were no known therapeutics.^2^ When the virus finally spread to Houston, Texas, area hospitals were better prepared, having benefitted from the experiences of New York, Seattle, and Europe. Houston Methodist, an eight-hospital healthcare system originally established at the height of the Spanish flu, played a key role in coordinating with other Texas Medical Center (TMC) hospitals to marshal their collective resources in the fight against the pandemic.

**Figure 1:**
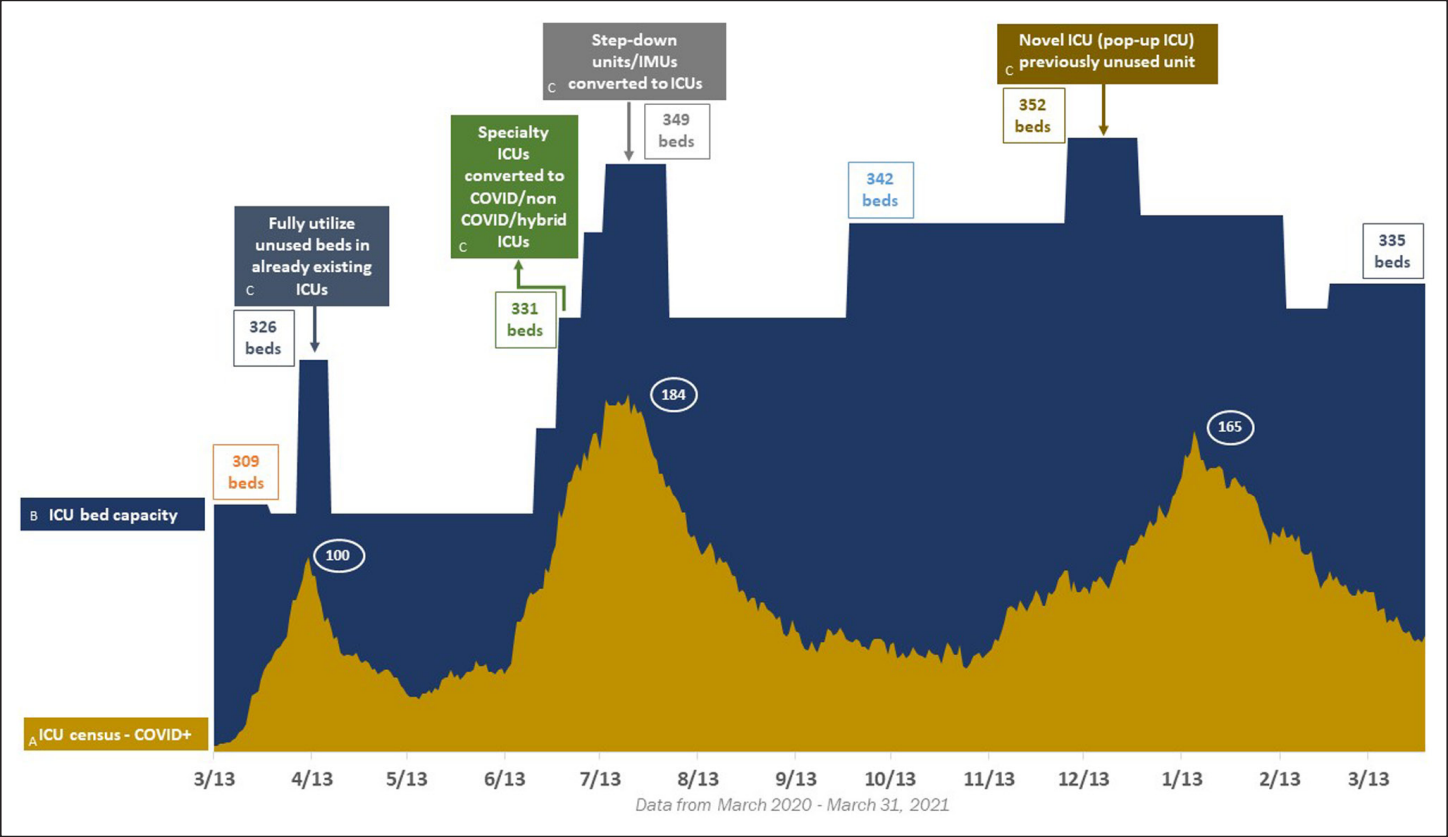
Houston Methodist intensive care unit (ICU) COVID-19 census, bed capacity, and bed types. (A) The peak ICU COVID-19 census was 184. (B) ICU bed capacity jumped from 309 beds before COVID-19 to 352 during infection surges. (C) Bed types included COVID-19–positive patients, COVID-19–negative patients, and hybrid beds. During the first surge, Houston Methodist suspended all elective, nonurgent surgeries in an effort to reduce demand for critical care beds. In May 2020, all surgeries and procedures resumed. The peaks that followed required increasing ICU bed capacity to accommodate COVID-19–positive patients as well as postoperative patients.

One of our first responses to the outbreak was to expand ICU bed capacity since approximately 5% of patients with severe COVID-19 required intensive care.^3^ As patient volumes surged, we rapidly increased our ICU capacity from 309 beds to more than 350 beds systemwide (***Figure 1***).

**Figure 2:**
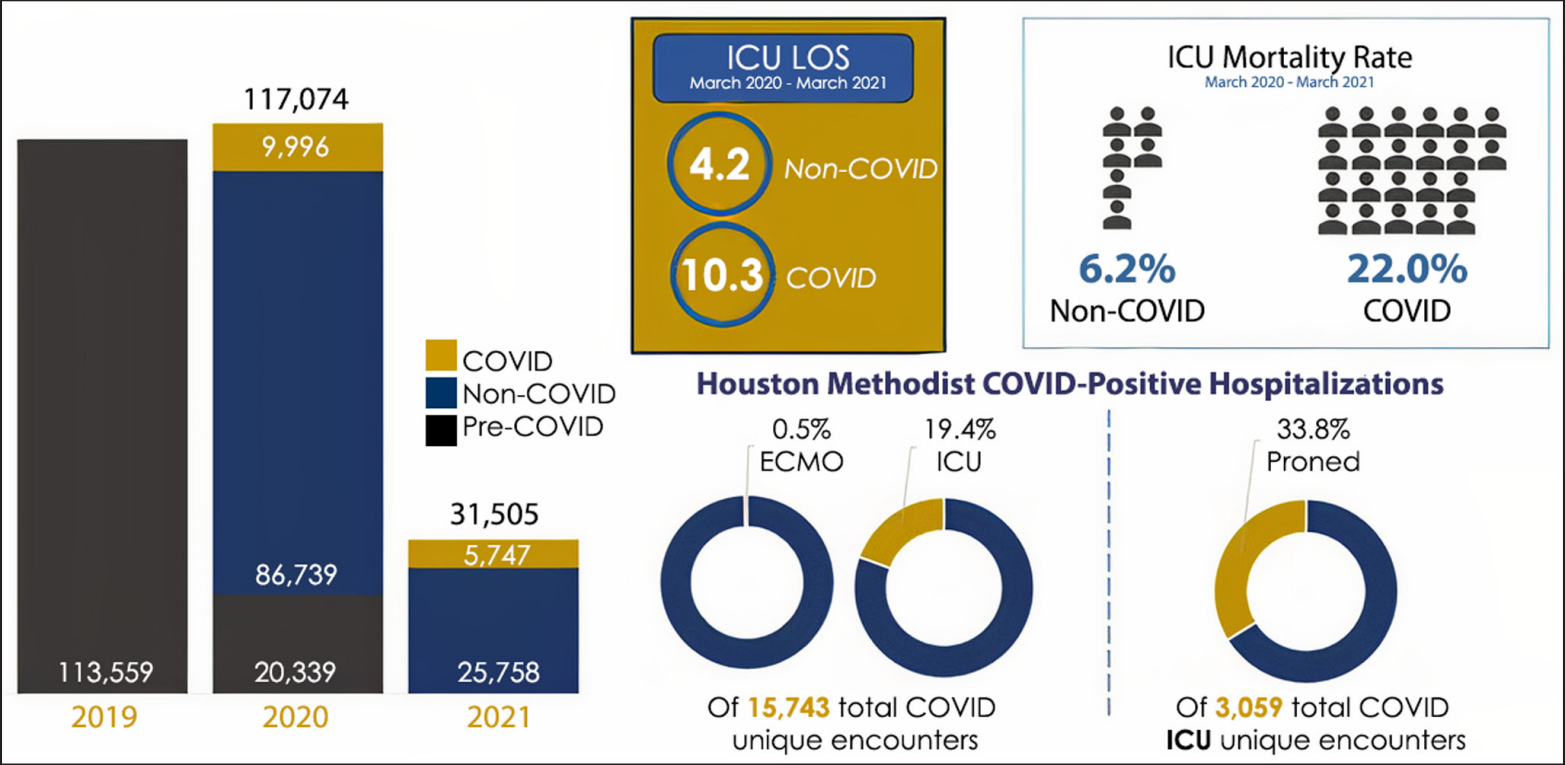
Houston Methodist COVID-19 outcomes. ICU: intensive care unit; LOS: length of stay.

During the consecutive surges of COVID-19 infections, patient profiles varied substantially. The first surge saw older patients with a higher comorbidity burden and higher ICU resource utilization, while the following peaks included younger patient populations with an overall lower comorbidity burden, ICU admission rate, and in-hospital mortality.^4^ Higher patient volumes with higher-acuity patients also exacted higher ICU resource utilization. Incoming referrals to ICUs combined with a limited availability of escalating support mechanisms such as extracorporeal membrane oxygenation (ECMO) further strained ICU operations. ***Figure 2*** shows the pandemic’s impact on our institution’s key metrics.

**Figure 3:**
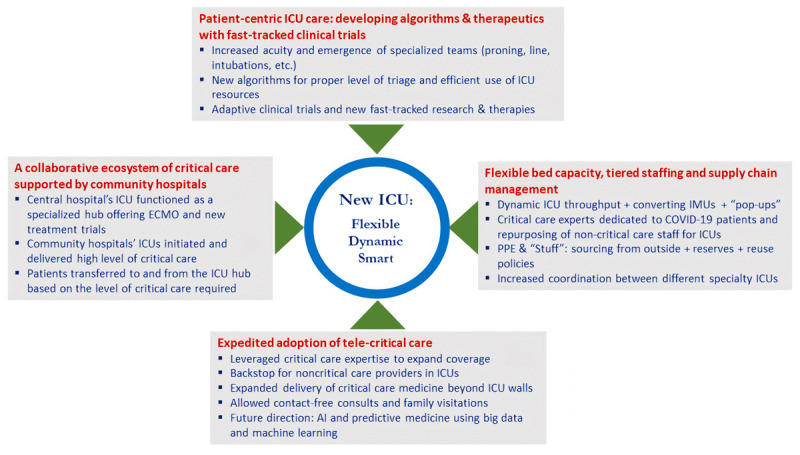
Key forces changing the face of the intensive care unit (ICU). ECMO: extracorporeal membrane oxygenation; IMU: intermediate care unit; PPE: personal protective equipment; AI: artificial intelligence.

Although existing hospital resources for critical care can meet general operating needs, they fall significantly short of the levels required for a pandemic. We responded to pandemic stressors by constantly adjusting workflows and ICU policies to efficiently deploy resources for timely care of critically ill patients. To avoid under- or overutilization of the ICU, specific triage workflows were developed to correctly identify patients with severe time-sensitive conditions and prioritize them over those with less urgent needs.^5^ Similarly, the homogeneity of COVID-19 management resulted in treatment protocols that were best administered through specialized teams, such as intubation, central line, and prone positioning teams. The key forces that shaped and transformed ICUs during COVID-19 are shown in ***Figure 3***.

## ICU Preparedness for Pandemic Surges

Multiple highly infectious disease outbreaks such as severe acute respiratory syndrome (SARS) in 2002,^6^ H1N1 influenza in 2009, Middle East respiratory syndrome (MERS) in 2012, and the periodic flare-ups of H5N1 virus^7^ have underscored the great need for pandemic preparedness for critical care providers. The Society of Critical Care Medicine’s (SCCM) Disaster Management Plan offered a practical blueprint for keeping ICUs safely operational through a pandemic.^8^ Our pandemic response closely followed SCCM’s guidelines by drawing on the following key levers.

### Strategic Planning and Incident Command Center

Houston Methodist established an incident command center to function as the epicenter for the overall institutional pandemic response.^9^ This housed a group of experts tasked with making daily operational decisions to ensure a safe environment for all patients and hospital staff while complying with state and local ordinances aimed at reducing the virus’ spread. Houston Methodist’s Center for Critical Care created planning and algorithm committees to standardize care and improve workflows for operations, logistics, laboratory diagnosis, infection control, and treatment protocols in all ICUs across the system. These protocols were formulated using a Delphi approach—taking into account updates in COVID-19–related research and guidelines from the National Institutes of Health, World Health Organization, and Infectious Disease Society of America—and included mechanical ventilation, ECMO, oxygen therapies, prone ventilation, intubation, tracheostomy, and cardiopulmonary resuscitation.^10,11,12,13,14,15,16,17,18,19,20,21,22,23,24^ Hospitals within the TMC convened regularly to coordinate allocation of resources, especially ECMO and drugs such as remdesivir, under emergency use authorization (EUA) apportioned by the federal resources for the region.

As businesses and communities locked down in response to the outbreak, TMC hospitals suspended all elective surgeries, which preserved resources for critical care. To prepare ICUs for the expected surge in COVID-19 patients, our incident command center focused on (1) expanding ICU bed capacity, (2) increasing ICU staffing, and (3) providing adequate provisions of personal protective equipment (PPE) and other ICU requirements such as ventilators, intravenous (IV) fluids, vasopressors, sedatives, neuromuscular blockade, and noninvasive oxygen devices.

### Expanding ICU Bed Capacity

Our ICU capacity was expanded in stages, with the initial increase coming from unused ICU beds.^25,26,27,28^ Bed capacity was then ramped up by improving ICU throughput: ICU patients were assessed frequently, and those deemed to be noncritical were either moved to step-down units or the acute care floor or were discharged. Lower-acuity patients still requiring hospitalization were transferred to a 20-bed highly infectious disease unit (HIDU) to decongest the ICUs and step-down units across the system. During surges, the HIDU bed capacity expanded to a maximum of 44 beds, and cancellation of elective surgeries reduced the demand for postoperative ICU beds.

Once the existing bed capacity had been pushed to its limit, new ICU beds were converted from existing step-down units, and an entire “make-shift” ICU was created from a previously unused ICU. During peak infection rates, the ICUs were divided into COVID-19, non–COVID-19, and hybrid units that cared for both types of patients. A tele-critical care infrastructure (termed “virtual ICU” at Houston Methodist) provided unique leverage, augmenting staffing capacity to deliver critical care expertise across multiple ICUs simultaneously. Expansion of bed capacity is shown in ***Figure 1***.

### Increasing ICU Staffing

During the initial surge in the spring of 2020, we implemented an SCCM-recommended tiered staffing model for our ICUs in which all critical care clinicians were deployed to manage COVID-19 patients.^27,29,30^ With the shutdown of many service lines, temporarily non-working clinicians were cross-trained by critical care specialists to care for non–COVID-19 ICU patients. Whenever the critical COVID-19 cases spiked, the non–COVID-19 patients were cared for by non-critical care hospitalists, surgeons, cardiologists, and other specialty staff with support and oversight by critical care specialists and a virtual ICU. In addition, many institutions brought in outside clinicians and nurses, granting them emergency privileges on an expedited basis.

Despite the necessary and well-intended ICU staffing restructuring, the protracted COVID-19 pandemic took a heavy toll on the physical and mental well-being of healthcare workers (HCWs). As with SARS, clinicians working throughout the COVID-19 pandemic experienced immediate and long-term psychological stress, leading to higher incidences of depression and insomnia and further exacerbating the long-standing problem of physician burnouts in the ICU.^31,32,33^ Within the command center, a resilience subcommittee called “mini-command” was formed to address staff safety and burnout (***Table 1***).

**Table 1:** Summary of the initiatives introduced at Houston Methodist to improve staff resilience and decrease burnout. ICU: intensive care unit; PPE: personal protective equipment.


STAFF SAFETY INITIATIVES	DESCRIPTION

Communication	• Town hall meetings were held to address concerns of the healthcare workers.

Music therapy	• Music therapy was used to calm staff anxiety stemming from the pandemic.

Adopt-a-unit	• COVID ICU was adopted by a non-COVID unit for 6 weeks, with staff in the COVID units receiving miscellaneous gifts and notes of affirmation and gratitude.

Mindfulness training	• Guided meditation was offered through a virtual platform, creating a systemwide mindfulness pause.

Safe rooms	• Family rooms in the ICUs were converted into “safe” rooms where the staff could unwind and relax during their ICU shifts.

Peer support	• Behavioral experts were available for anyone who needed a mental health consultation. • A dedicated chaplain was available to educate physicians and staff on stress management skills and adaptive/maladaptive coping skills.

Protecting staff	• Adequate PPE and powered air purifying respirators (PAPRs) were available to all healthcare workers. • Virtual rounding was done through tele-ICU–enabled laptops outside patient rooms to mitigate the risk of transmission. • Staff underwent frequent testing for COVID-19 and had prioritized access to the COVID-19 vaccine.


### Procurement of PPE and Other ICU Supplies

During the height of the pandemic, the extraordinary demand for critical care services required a concerted and strategic coordination effort. Under normal circumstances, most facilities use just-in-time inventory management, with modest amounts of reserves for key materials. Except for government agencies, no institution has the capacity or budget to stockpile critical supplies. Hence, COVID-19 surges strained hospital systems across the country with severe shortages of PPE, particularly N95 respirators approved by the National Institute for Occupational Safety and Health.^34,35^ Fortunately, Houston Methodist continuously sources PPE as part of its ongoing operations, giving us enough local reserves during the initial outbreak. Even so, PPE consumption (or burn rate) in the ICUs was high, requiring a sterilization and reuse policy consistent with Centers for Disease Control and Prevention guidelines for the N95 masks.^36,37^ The reuse policy was also extended to isolation gowns during the peak surges. A stable supply of PPE and adequate inventory of ventilators and other key ICU materials, along with innovations such as isolation boxes and drug pump extensions, allowed our ICUs to operate daily without interruption. Our hospital also procured and rapidly deployed LUCAS, an automated chest compression device for use in cardiopulmonary resuscitation of COVID-19 patients. Use of this device limited the number of staff required during a code, thus reducing the risk of exposure to HCWs.^38^ At some institutions, limited supplies of lifesaving equipment ignited ethical dilemmas for HCWs when oxygen and ventilators had to be rationed, adding a moral burden to the already stressed ICU workers.

## ICU Operations and Patient Care During Covid-19 Pandemic

**Figure 4:**
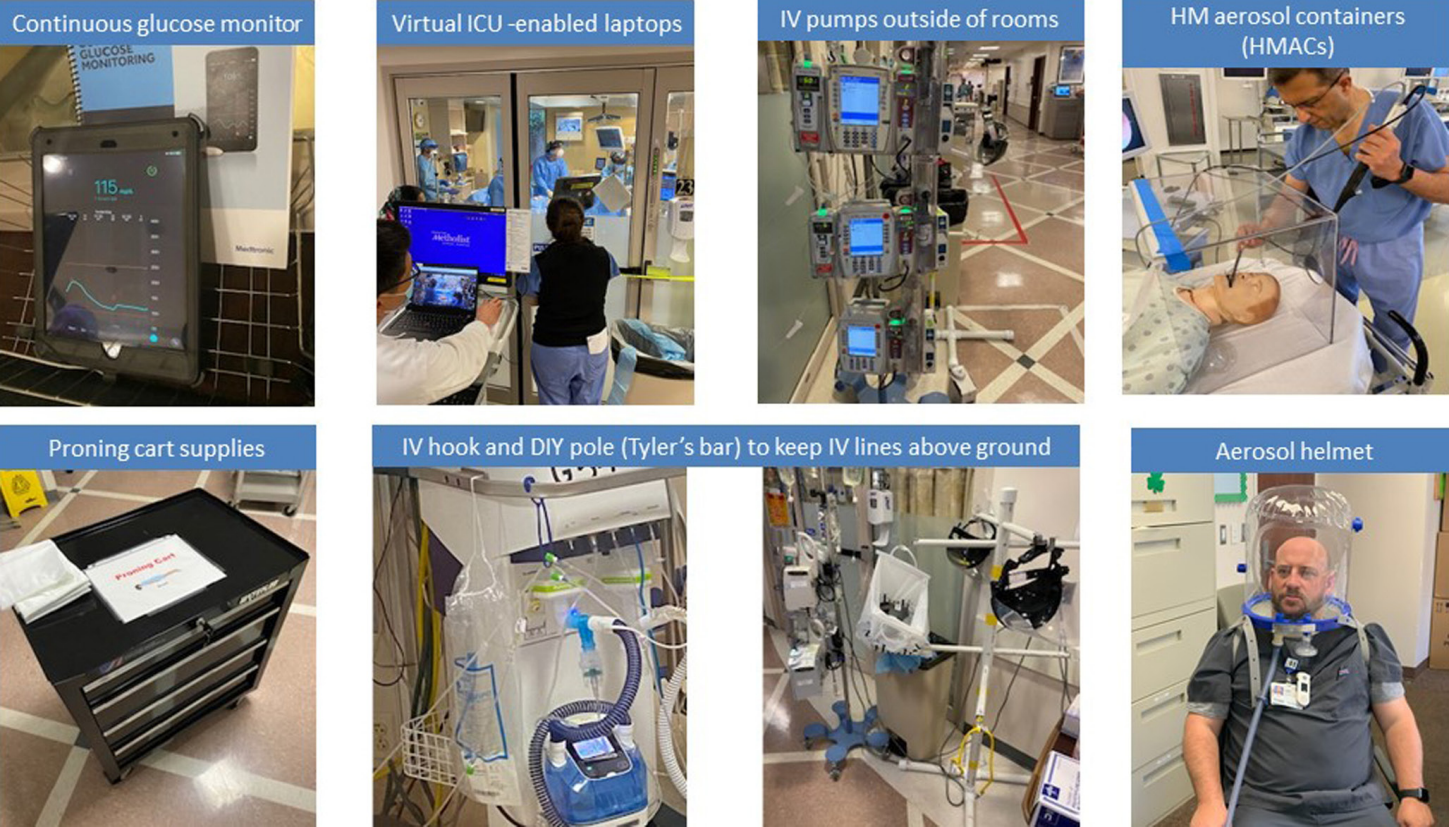
Intensive care unit (ICU) innovations generated by COVID-19. IV: intravenous; DIY: do it yourself.

We deployed all resources with institutional discipline to meet the needs of COVID-19 patients during the pandemic’s peaks and plateaus. The operations, protocols, and algorithms of ICUs required frequent modifications due to the influx of COVID-19 patients and increase in COVID-19–related complications. From triage adjustments to infection control to implementation of new technologies and therapeutics, ICUs across the country operated with an “all-hands-on-deck” approach, changing the face of ICUs in the process. ***Figures 3***, ***4*** show some of the key innovative responses deployed during the pandemic. In addition, the link provides a video overview of our ICUs’ response to the pandemic (see ***Video 1***).

**Video 1:** Overview of Houston Methodist intensive care unit response to the COVID-19 pandemic. *https://youtu.be/u1XAko0Ra9I*

### Triage Process

The key objective of the triage process is to identify patients with a deteriorating condition in a timely manner and prioritize them over those with less-severe conditions. Under-triage, or failure to capture patients with severe illness, contributes to delays in time-sensitive interventions and can lead to morbidity and mortality. Conversely, over-triage prioritizes patients with less-urgent presentations, thus diverting limited time and resources away from the patients most in need. In our ICUs, a key determinant of triage status was the ordinal scale of the patient, in this case defined by their oxygen level.^39^ During the first surge, initial guidelines supported early intubation, and use of noninvasive ventilation and high-flow oxygen was contraindicated given the concern for elevated risk of aerosolization and viral exposure to HCWs.^40,41,42,43,44^ This led to the overutilization of ICUs, mechanical ventilators, and other critical care resources. Later studies demonstrated that the risks were no higher to HCWs if better-fitting noninvasive ventilation masks were used for patients in addition to the use of negative pressure rooms, a lesson we applied in our ICUs.^45^

## ECMO: Optimizing Utilization of a Limited Resource

During the 2009 H1N1 influenza epidemic, ECMO—a salvage therapy for severe acute respiratory distress syndrome (ARDS)—yielded promising results, albeit with the attendant risk of complications such as bleeding and nosocomial infections. In the last decade, use of ECMO has grown, supported by results from randomized trials.^46,47,48^

In the early phase of the pandemic, Extracorporeal Life Support Organization (ELSO) guidelines recommended ECMO for eligible COVID-19 patients with severe cardiopulmonary failure.^49^ However, the number of COVID-19 patients with severe ARDS who required ECMO was unknown. The administration of such complex therapies during a pandemic was challenging, requiring multidisciplinary planning, careful triage and resource allocation, and appropriate personnel training combined with strict infection control measures. With a well-established ECMO program and experienced staff, our hospital quickly became one of the specialized centers, managing more than 70 COVID-19 patients requiring ECMO.

**Figure 5:**
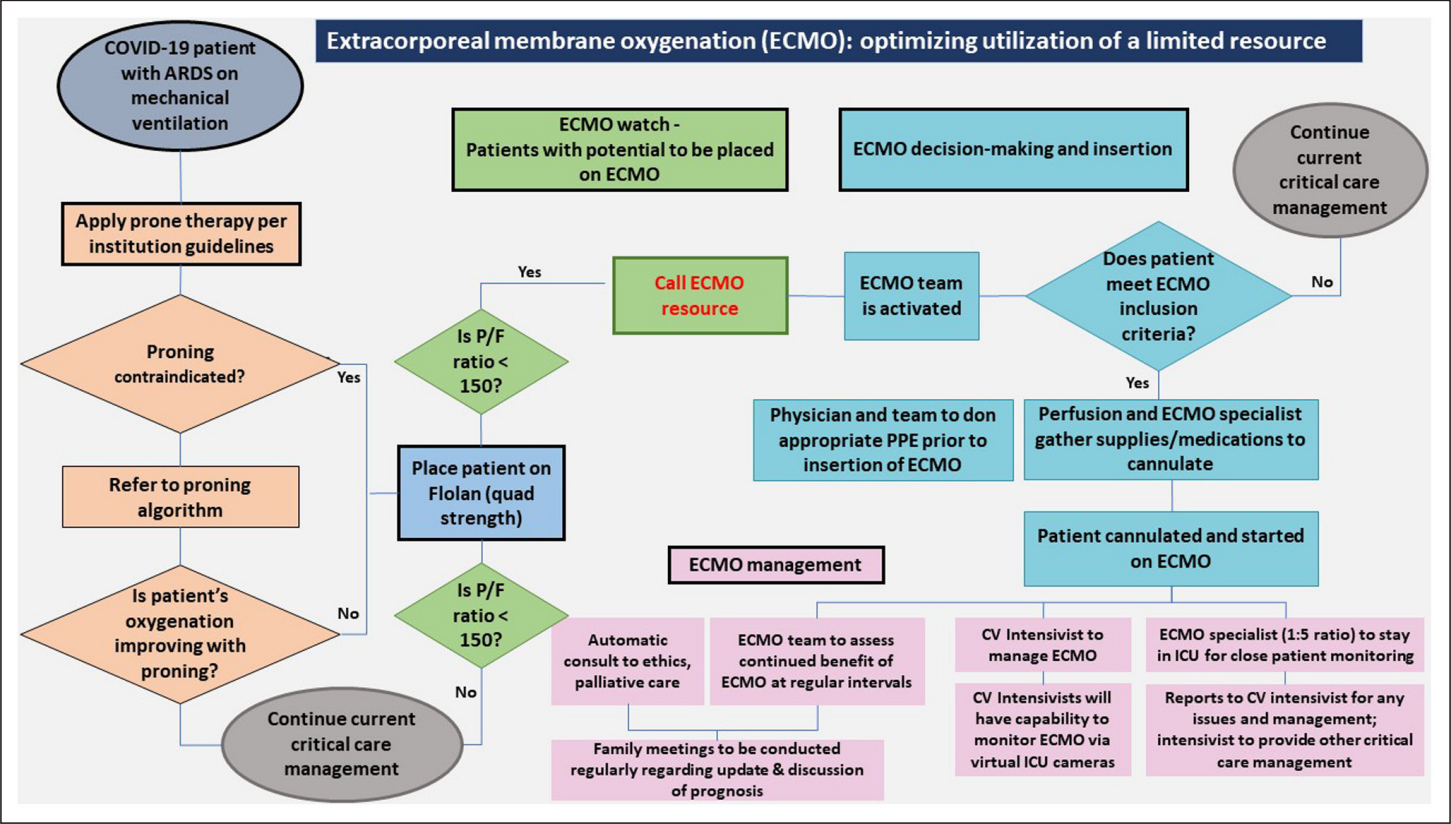
Triage algorithm for extracorporeal membrane oxygenation (ECMO) utilization in COVID-19 patients with acute respiratory distress syndrome (ARDS). (1) COVID-19 patients with ARDS are placed on mechanical ventilation adhering to institutional guidelines for ARDS management. (2) Prone therapy is strongly recommended in all patients with moderate to severe ARDS and should be performed if the patient’s PaO_2_/FiO_2_ (P/F) ratio is < 150. These patients should also be considered for pulmonary vasodilator therapy, especially if proning is contraindicated or the patient’s oxygenation does not improve or worsens with prone therapy. (3) Despite these interventions, if the patients’ P/F ratio is still < 150, these patients should be placed on ECMO watch. (4) The ECMO team consisting of cardiovascular (CV) intensivists, CV surgeon, and ECMO specialist uses a multidisciplinary approach based on current guidelines to determine whether a patient should be placed on ECMO. If so, perfusionist and ECMO specialists prepare supplies and medications for cannulation. The patient is then cannulated by a CV surgeon. Institutional guidelines are followed for the appropriate donning of PPE prior to insertion of ECMO. (5) ECMO is managed by the CV intensivist. An ECMO specialist is stationed in the ICU for close patient monitoring. All issues are directed to the CV intensivist, who can monitor ECMO patients via the virtual ICU cameras. Automatic ethics and palliative care consults are generated for every ECMO patient. The ECMO team continues to assess the benefits of ECMO on a regular basis and holds family meetings regularly. (6) The critical care intensivist continues to manage all other critical care aspects of the patient and work closely with the ECMO team. ARDS: acute respiratory distress syndrome; PPE: personalized protective equipment; CV: cardiovascular; ICU: intensive care unit.

During each surge, triage decisions and patient selection criteria for ECMO in the ICUs remained dynamic, depending on the available resources, patient load, and capacity at the time of referral. Best practices guidelines called for early ECMO use if a patient failed to improve despite optimal use of ARDS strategies such as lung-protective ventilation, prone ventilation, and pulmonary vasodilators. Higher age, immunocompromised status, duration of mechanical ventilation prior to ECMO, associated extrapulmonary infection, low respiratory compliance, and noninfluenza diagnosis are some of the main determinants of poorer outcomes.^50^ The exclusion criteria for age among non–COVID-19 ECMO patients was < 80 years; however, with guidance from ongoing studies, ECMO use for COVID-19 patients was restricted to < 70 years of age during the first surge. The age criterion was revised in subsequent surges based on prediction models and risk-adjusted data that showed lower survival for older patients.^51,52^ ***Figure 5*** describes the triage algorithm created for ECMO utilization in COVID-19 patients with ARDS.

### Adjusting ICU Space for Aerosol Infection Risk

Given the high aerosolized infection risk of COVID-19, the need for airborne infection isolation rooms or negative-pressure rooms far exceeded the number of rooms available. To protect the staff, high-efficiency particulate air purifiers were placed in each patient room to reduce aerosol spread. With the single-room setup in our COVID-19 and hybrid ICUs, a novel yet effective risk-mitigation method was to move infusion pumps, detachable ventilator monitors, and continuous renal replacement therapy equipment outside of the room and use IV extensions. These strategies not only decreased staff exposure but also reduced our daily PPE consumption by more than one-third.^53^ Another innovative approach to mitigating aerosol infection risk included the use of helmet-based ventilation that reduced particle dispersion and air leaks. Compatible with multiple oxygen sources, helmet-based ventilation performed better than the full-face bilevel positive airway pressure and continuous positive airway pressure masks that were sometimes ill fitting and resulted in higher aerosolization.^54^

### Relaxed Regulations, Novel Therapies, and Innovative Tools

The rapid transmission of SARS-CoV-2 combined with the urgency to find an effective treatment required a flexible, fast-track approach to the development of therapeutics instead of the conventional approach using long-term randomized controlled trials.^55^ During 2020, the majority of therapies were repurposed from an existing treatment for other indications, such as remdesivir, which was initially used for Ebola. To encourage research and development of new therapies, the US Food and Drug Administration (FDA) created a special emergency program for possible coronavirus therapies called the Coronavirus Treatment Acceleration Program, enabling clinicians to gain valuable knowledge about the safety and efficacy of new treatments. In addition, the FDA used its EUA to authorize the use of therapeutics that had not undergone the usual rigorous testing, allowing clinicians to administer several experimental therapies.^56^ An important therapeutic administered early in the pandemic was convalescent plasma, a treatment that was used for the Spanish Flu in 1918 and more recently during the SARS, MERS, and Ebola outbreaks. However, early data supporting the efficacy of plasma therapy for COVID-19 patients were derived entirely from nonrandomized trials and case series. We were the first hospital in the United States to administer this therapy to a patient with severe COVID-19 disease on March 28, 2020, under EUA.^57,58^

The pandemic experience suggests that research studies designed on adaptive trial platforms were more efficient in evaluating a number of therapies concurrently through multiple treatment arms using common controls, which in turn would seamlessly transition to next-phase studies generating faster results. To fast-track potential therapies and pivotal research, Houston Methodist Research Institute and its Institutional Review Board were able to establish several NIH/National Institute of Allergy and Infectious Diseases adaptive trials as well as initiating its research in epidemiology, therapeutics, and vaccines.

Many COVID-19 patients developed hyperglycemia due to critical illness and frequent glucocorticoid use or had diabetes as a comorbidity, requiring IV insulin infusion along with hourly glucose monitoring to prevent hypo- or hyperglycemia. We piloted a continuous glucose monitoring device that tracked blood glucose levels continuously in real time, thereby reducing the need for frequent bedside glucose testing and the corresponding risk of exposure to nursing staff who otherwise had to enter patients’ rooms hourly for finger-stick glucose checks.^59^

## Pivotal Role of Tele-Critical Care

Tele-critical care platforms provide remote monitoring and treatment of ICU patients while extending access to critical care physicians and registered nurses along with many decision-support tools necessary for ICU care. Prior to the pandemic, our system had installed an audio/visual communication infrastructure in each ICU room to enable remote delivery of critical care services. The launch of this program, named virtual ICU or vICU, coincided serendipitously with the outbreak. The exigencies of the pandemic expedited the vICU roll out, which at its full operational capacity allowed the hospital to successfully implement a tiered staffing model.^27^ The vICU physicians and critical care nurses augmented patient care provided by bedside clinicians and nurses for COVID-19 patients in the ICUs and step-down units while also supporting noncritical care clinicians working with the non–COVID-19 ICU patients. For patients in beds where vICU infrastructure was lacking, mobile vICU carts were deployed. In addition, the vICU infrastructure connected family members with COVID-19 ICU patients for a virtual family visit and enabled remote consultation with a specialist.^60^ ***Table 2*** shows the many contributions of a virtual ICU during COVID.

**Table 2:** Virtual intensive care unit (vICU) contributions to a changing ICU during COVID. PPE: personal protective equipment; CV: cardiovascular; ECMO: extracorporeal membrane oxygenation.


CHALLENGE	CONTRIBUTION

Shortage of PPE	• Enabled contact-free consults where specialists use vICU cameras to assess ICU patients • Positioned local vICU-enabled laptops outside patient rooms in the COVID-19 ICUs to eliminate the need for donning and doffing of PPE

Restricted family visitation	• Used vICU infrastructure to implement remote family visitation program

Shortage of staff and ICU beds	• Increased staffing and bed capacity by deploying virtual critical care physicians and nurses to augment bedside ICU clinicians • Provided oversight of non–critical-care providers during peak of pandemic when critical care specialists were assigned to manage COVID-19 patients

ECMO support	• Used vICU-enabled laptops to allow communication between CV intensivists outside the ICU and CV surgeon/ECMO team operating inside to limit essential personnel


## Role of Community Hospitals

With more than 350 ICU beds systemwide, our hospitals play an important role in the delivery of critical care services within their local communities that make up the greater Houston metropolitan area. Since the outbreak of COVID-19, the system hospital ICUs have evolved symbiotically, learning from each other, exchanging valuable information and guidelines, and creating a collaborative ecosystem of critical care medicine under the umbrella of Houston Methodist Center for Critical Care.

The close collaboration between HM’s community hospitals and its central campus during the pandemic extended in particular to the research and clinical trials for COVID-19 therapeutics. COVID-19 patients in our community hospitals were routinely screened for eligibility in the available trials and were transferred to the main hospital if (A) they met the study’s inclusion criteria, (B) they were accepted by the principal investigators, and (C) they were able to provide consent. For some of the higher acuity patients, community hospitals initiated ECMO cannulation until they could be transferred to the main hospital for a higher level of care.

## Discussion

The COVID-19 pandemic has had a profound impact on the healthcare industry and on ICUs around the world. More than 700,000 people have died from COVID-19 in the US alone, many of them in hospitals.^61^ Of them, more than 3,600 US healthcare workers died in the line of duty while caring for COVID-19 patients during the pandemic’s first year.^62^ The healthcare industry also faced a social media “infodemic”—a proliferation of fake news and misinformation that diluted the legitimate global efforts to combat a highly infectious disease.

Despite the massive coordination of resources, the experience of all ICUs worldwide has not been and cannot be expected to be uniform. ICUs in many regions have faced decidedly different levels of infection risk, with different approaches to treatment and different outcomes. During the pandemic, certain exogenous factors have had a varying impact on ICU operations. First, the severity of the outbreak differed by region, exacting different levels of stress on local ICUs. Second, resource limitations and socioeconomic inequities have greatly hampered the ability of ICUs in different regions to follow the prescribed response to a pandemic. Third, the politicization of healthcare policy and debasement of scientific facts have contributed to vastly different infection and mortality rates across the globe. While our experience may not be directly applicable to ICUs in certain parts of the world, it provides significant guidance to the approach of critical care during a pandemic.

Since the onset of the COVID-19 pandemic, the field of critical care medicine has undergone significant changes that are likely to endure for years to come. ICUs have become more flexible, dynamic, and responsive to the demands of critical care medicine. The pandemic expedited the adoption of tele-critical care medicine and pushed ICU operations to be more efficient in staffing and resource utilization. It also provided an opportunity to test and adapt the disaster planning response of each hospital system. From virtual family visitation to placement of IV pumps outside patient rooms, necessity and innovation have served as dual catalysts for positive change.

## Key Points

- The COVID-19 pandemic required strategic planning and rapid execution to increase bed capacity and staffing while ensuring an uninterrupted supply of personal protective equipment and other critical materials needed in intensive care units (ICUs).
- To accommodate the surge of COVID-19 patients, ICUs expanded bed capacity by using existing unused bed capacity and creating new ones. The Society of Critical Care Medicine’s tiered staffing model allowed hospitals to leverage critical care specialists to support a higher volume of ICU patients during peak surges.
- Tele-critical care enabled the delivery of timely critical care services beyond the traditional boundaries of the ICU.
- Houston Methodist’s central hospital campus and community hospitals worked in tandem to create a collaborative ecosystem that delivered a high level of critical care, including specialized care such as extracorporeal membrane oxygenation.
- ICUs participated in adaptive clinical trials and other fast-track research to develop new therapeutics and innovative approaches to mitigate aerosol infection risk.
